# Supplementary material for: Synthetic Peptides as a Promising Alternative to Control Viral Infections in Atlantic Salmon
Source: Pathogens. 2020 Jul 23;9(8):600. doi: 10.3390/pathogens9080600 (PMC7459813; doi:10.3390/pathogens9080600)
Supplement: Supplementary file 1 [file pathogens-09-00600-s001.zip › pathogens-839765/Supplementary_material_839765_R2.docx]

Synthetic peptides as a promising alternative to control viral infections in Atlantic salmon

Constanza Cárdenas , Fanny Guzmán, Marisela Carmona, Cristian Muñoz, Luis Nilo, Alvaro Labra and Sergio H. Marshall

**Supplementary Material**

**Table S1.** Structural assessment for the homology models of IPNV VP2 homotrimer, and ISAV RdRP subunits PA, PB1 and PB2. The analysis made through the PDBsum server is presented, with the percentage of residues found in allowed and disallowed regions according to the Ramachandran plot. The ProSa server was also used, and a graph is presented with the location of each model within the available structures, and their respective Zscore.

| Virus | Protein | PDBSum-Ramachandran | | | | Prosa |
| --- | --- | --- | --- | --- | --- | --- |
|  |  | **Allowed** | | | **Disallowed** |  |
| **IPNV** | **VP2*** | **80.3%** | **15.2%** | **2.1%** | **2.4%** | **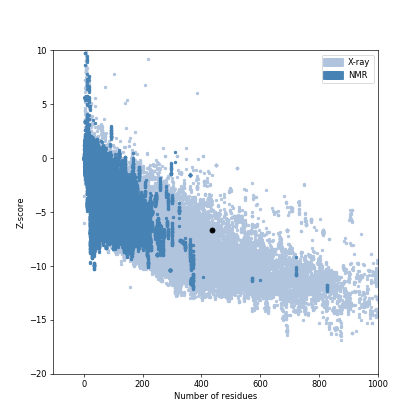Zscore=-6.66** |
| **ISAV** | **PA** | **66.3%** | **25.5%** | **5.7%** | **2.5%** | **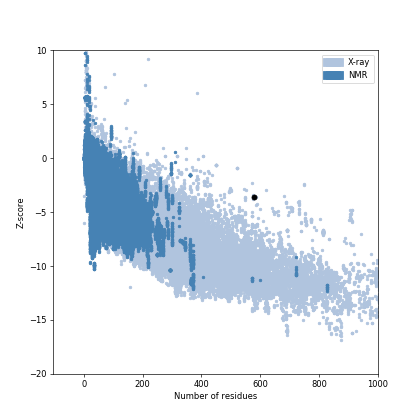Zscore=-3.62** |
|  | **PB1** | **76.2%** | **19.6%** | **2.7%** | **1.6%** | **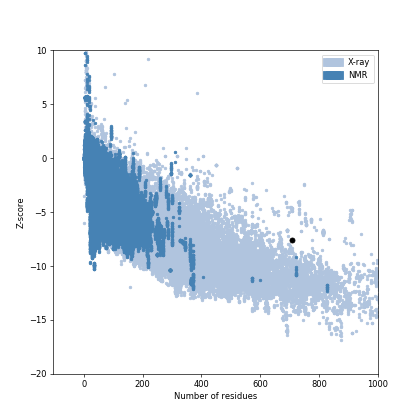Zscore=-7.56** |
|  | **PB2** | **65.2%** | **26.3%** | **5.4%** | **3.0%** | **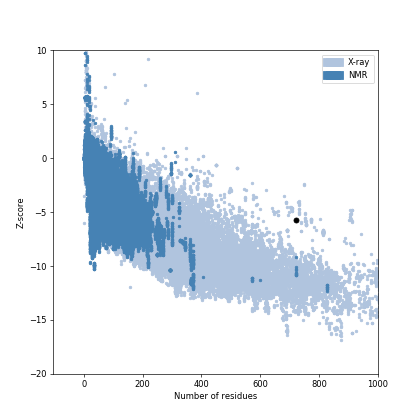Zscore=-5.69** |

***Homotrimer**


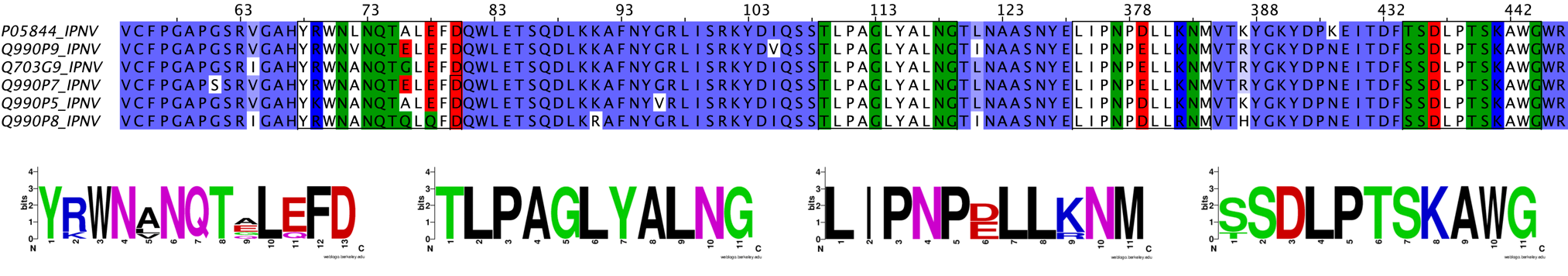


**Figure S1.** Multiple Alignment of the VP2 protein form IPNV strains Jasper, Ab, Sp., Te, C2, and He (Uniprot accession P05844, Q990P9, Q703G9, Q990P7, Q990P5, Q990P8 respectively). Regions selected to synthesize are indicated, and below the alignment the logos [1] of the sequences showing the conservation between IPNV genogroups.


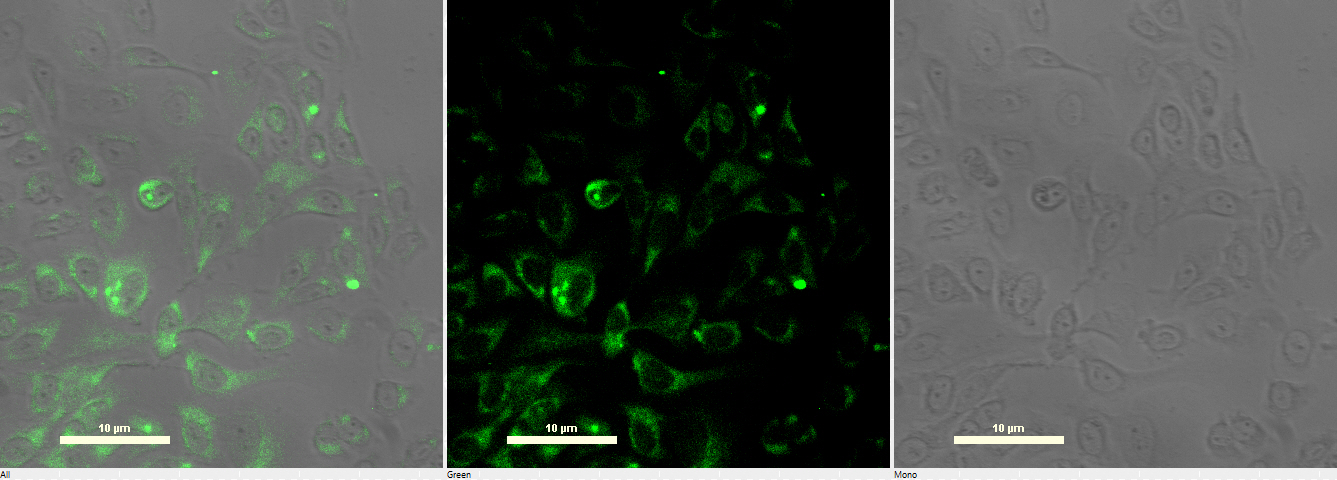


**Figure S2.** Association of GIM182 peptide to the CHSE-214 cells by fluorescence microscopy with the peptide recognized by a specific antibody. From right to left: the bright field, the antibody recognition and both filters at the same time

**Figure S3.** Preliminary assays with the peptides designed against IPNV VP2 trimer. Significance values by two way ANOVA at P <0.01 * and P> 0.001 **. Error bars represent standard deviation of measurements in triplicate.

**Table S2.** Statistical analysis for the comparison of survival curves. Analysis was made with Log-rank (Mantel-Cox) test

|  | **All curves** | **C virus vs T1** | **C virus vs T2** | **C virus vs C peptide** | **C peptide vs T1, T2** |
| --- | --- | --- | --- | --- | --- |
| **Chi square** | 17.8 | 12.03 | 4.14 | 12.8 | 3.23 |
| **df** | 3 | 1 | 1 | 1 | 2 |
| **P value** | < 0.0005 | 5E-04 | 0.04 | 0 | 0.2 |
| **P value summary** | *** | *** | * | *** | ns |
| **Significant difference** | Yes | Yes | Yes | Yes | No |

**Table S3.** GenBank accession numbers for the ISAV RdRp subunits used in the multiple alignments and comparison.

| **Isolate** | **PB2** | **PB1** | **PA** |
| --- | --- | --- | --- |
| SK779 | ABW93482 | ABW93483 | ABW93485 |
| 752 | ADF36495 | ADF36496 | ADF36498 |
| 909 | ADF36505 | ADF36506 | ADF36508 |
| 909-1 | AIW00348 | AIW00349 | AIW00351 |
| ISAV1 | ABG65741 | ABG65755 | ABG65783 |
| ISAV2 | ABG65742 | ABG65756 | ABG65784 |
| ISAV3 | ABG65743 | ABG65757 | ABG65785 |
| ISAV4 | ABG65744 | ABG65758 | ABG65786 |
| ISAV5 | ABG65745 | ABG65759 | ABG65787 |
| ISAV6 | ABG65746 | ABG65760 | ABG65788 |
| ISAV7 | ABG65747 | ABG65761 | ABG65789 |
| ISAV8 | ABG65748 | ABG65762 | ABG65790 |
| ISAV9 | ABG65749 | ABG65763 | ABG65791 |
| ISAV10 | ABG65750 | ABG65764 | ABG65792 |
| ISAV11 | ABG65751 | ABG65765 | ABG65793 |
| Vir22 | ABG65754 | ABG65768 | ABG65796 |
| Vir25 | ABG65753 | ABG65766 | ABG65794 |
| Vir28 | ABG65752 | ABG65767 | ABG65795 |
| Glesvaer | ADR77505 | ADR77506 | ADR77506 |
| RPC-NB0 | AAX56293 | ABF68026 | ABF68029 |


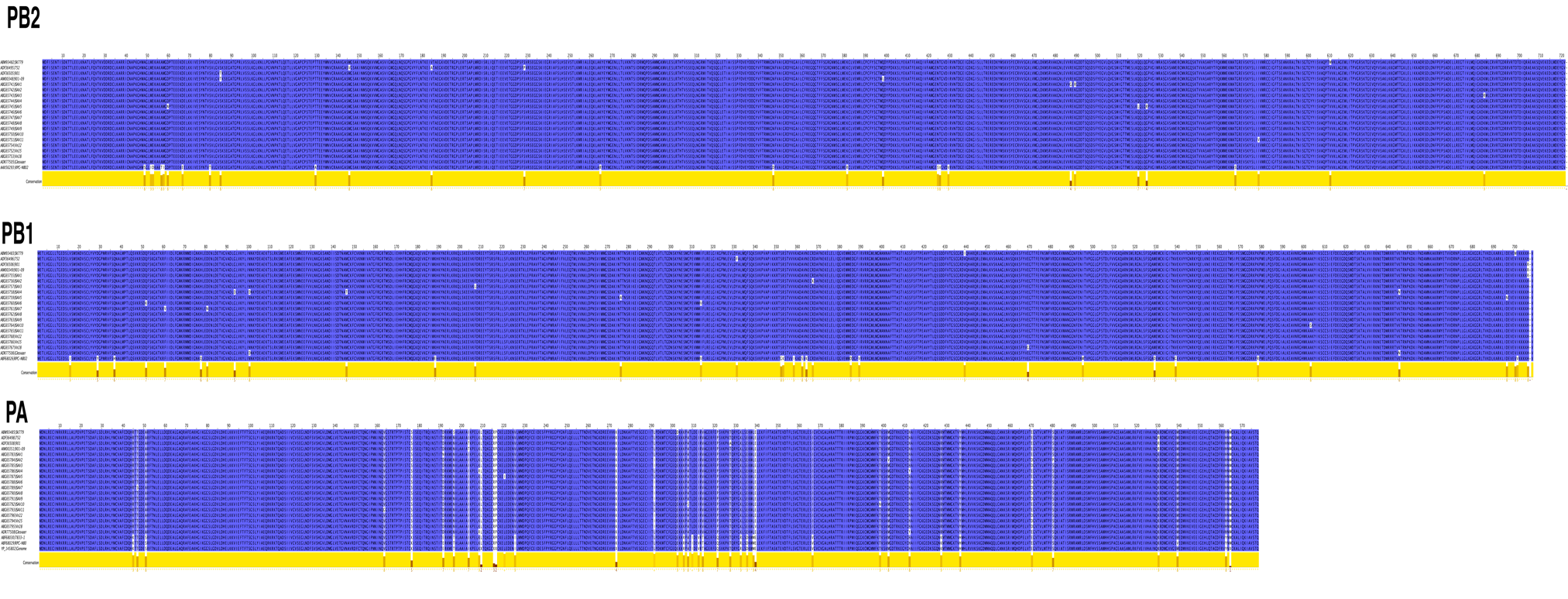


**Figure S4.** Scheme of the multiple alignment of the subunits of the RdRP of ISAV. The color indicates the degree of conservation, as well as the yellow bar under the sequences.


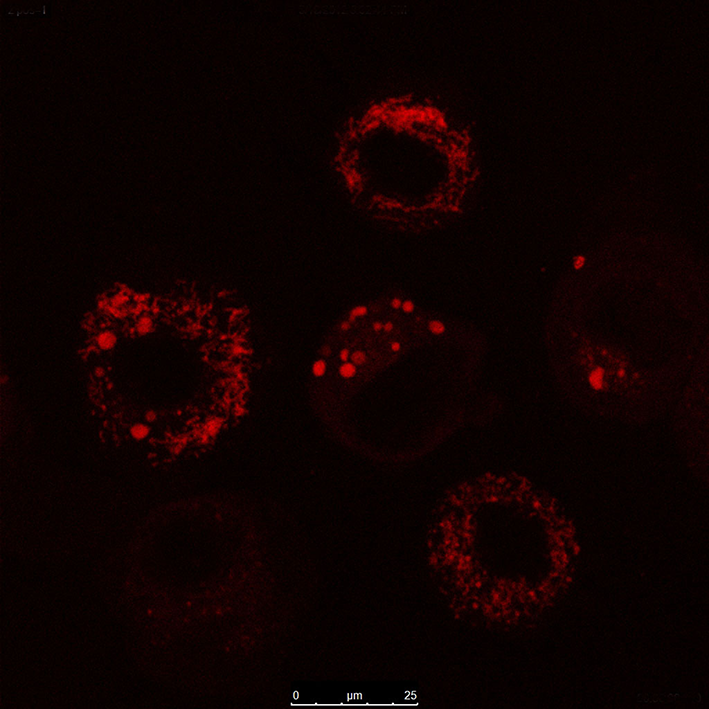


**Figure S5.** SHK-1 cell association of peptide GIM538 with rhodamine by confocal microscopy.

**Figure S6.** Preliminary in vitro test for the peptides against ISAV RdRp. Peptides were tested at two different concentrations at 12 and 24 hours. Fold change was measured with ELF 1α as host gene. Error bars represent standard deviation of measurements in triplicate.

**Table S4.** Primers used for RT-PCR analysis: IPNV VP1 and VP2 proteins; ISAV segment 6 and segment 8 vRNA amplification and ELF 1 α host gene.

| **Virus** | **Gen** | **Primer** | **Sequence** |
| --- | --- | --- | --- |
| IPNV | VP1 | VP1SNP-F | CTGGTCCAGAAACCCTAAGAC |
|  |  | VP1SNP-R | GTGTGTATCTCTCCCCTTTTGG |
|  | VP2 | VP2SNP-F | CAACAGGGTTCGACAAACCATAC |
|  |  | VP2SNP-R | TTGACGATGTCGGCGTTTC |
| ISAV | Segment 6 | vRNAS6-F | TGCAGCATTCATGTACAAGTC |
|  | Segment 8 | vRNAS8-F | GATGGGGATCAACAGGGAAA |
|  | ELF 1α | ELF 1α-F | CCCCTCCAGGACGTTTACAAA |
|  |  | ELF 1α-R | CACACGGCCCACAGGTACA |

**References**

1. Crooks, G.E. WebLogo: A Sequence Logo Generator. *Genome Res.* **2004**, *14*, 1188–1190.
